# Above the Matrix: Functional Roles for Apically Localized Integrins

**DOI:** 10.3389/fcell.2021.699407

**Published:** 2021-08-13

**Authors:** Raven J. Peterson, Michael Koval

**Affiliations:** ^1^Division of Pulmonary, Allergy, Critical Care and Sleep Medicine, Department of Medicine, Emory University School of Medicine, Atlanta, GA, United States; ^2^Department of Cell Biology, Emory University School of Medicine, Atlanta, GA, United States

**Keywords:** apical/basolateral polarity, tight junctions, cell migration, mechanosensing, cytoskeleton

## Abstract

Integrins are transmembrane proteins that are most typically thought of as integrating adhesion to the extracellular matrix with intracellular signaling and cell regulation. Traditionally, integrins are found at basolateral and lateral cell surfaces where they facilitate binding to the ECM and intercellular adhesion through cytosolic binding partners that regulate organization of actin microfilaments. However, evidence is accumulating that integrins also are apically localized, either endogenously or due to an exogenous stimulus. Apically localized integrins have been shown to regulate several processes by interacting with proteins such as connexins, tight junction proteins, and polarity complex proteins. Integrins can also act as receptors to mediate endocytosis. Here we review these newly appreciated roles for integrins localized to the apical cell surface.

## Introduction

Integrins are classically thought of as mediating intercellular interactions and binding to the extracellular matrix (ECM), so their role in cell adhesion is well characterized (Hynes, 2002; Geiger and Yamada, 2011). Integrin mediated adhesion regulates the actin cytoskeleton, enabling mechanosensation, by coordinating cell responses to force transmitted from the extracellular environment (Brakebusch and Fassler, 2003; Schwartz, 2010; Weber et al., 2011; Li and Springer, 2017). Integrins are linked to the actin cytoskeleton via scaffold proteins (including talin, kindlin, and paxillin) which also form signaling hubs that facilitate intracellular signaling (Sun et al., 2019; Kadry and Calderwood, 2020). Signaling through integrins is unique in that it is bidirectional, as it can be initiated by external ligand binding (outside-in signaling) or by the interactions with cytosolic scaffold proteins (inside-out signaling) (Hynes, 2002; Ye et al., 2012; Humphries et al., 2019; Kadry and Calderwood, 2020).

Much of what is known about integrins and their function comes from studying cell-cell interactions between leukocytes (Chigaev and Sklar, 2012; Martin-Cofreces et al., 2018) and cell/ECM adhesion localized to basolateral cell surfaces (Schwartz, 2010; Geiger and Yamada, 2011). However, there is a growing literature demonstrating that integrins are present at the apical surface of polarized cells. Examples of functional roles for apically localized integrins in polarized epithelial and endothelial cells have been reported in a variety of fields yet they remain under studied. Unlike integrins that organize focal adhesions or cell-cell interactions, apical integrins tend to be dispersed throughout the plasma membrane which makes assigning them to specific structures or molecular complexes challenging.

In this review we describe examples of apically localized integrins that exist in polarized cells and have been linked to functional roles. We first discuss integrin structure and antibodies that can detect and influence integrin activation state followed by a summary of intracellular trafficking pathways used by newly synthesized integrins in polarized cells. We then review demonstrated roles for apical integrins ranging from wound repair and mechanosensing to epithelial barrier function. The implications of these roles for apical integrins in normal and pathological cell behavior is examined, along with future directions.

## Integrin Structure and Conformation

**FIGURE 1:**
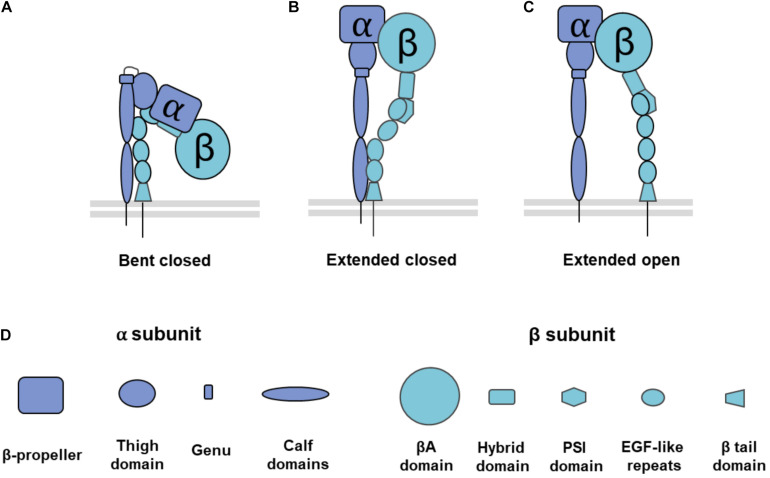
Overview of integrin conformation and domain composition. Integrins can adopt a range of conformation structures from **(A)** bent closed conformation that is inactive with a low affinity for external ligands, to **(B)** extended closed conformation, an intermediate conformation in that is active but has a low affinity for external ligands, and **(C)** extended open conformation that is active with a high affinity for external ligands. **(D)** Structural domains of each integrin subunit. Integrins α1, α2, α10, α11, αD, αE, αL, αM, and αX have an I-domain in their β-propeller subunit. PSI: Plexin, Semaphorin, Integrin domain, EGF: Epidermal Growth Factor domain. Adapted from Luo et al. (2007) and Campbell and Humphries (2011).

Integrins are single pass transmembrane glycoproteins found as obligate α/β heterodimer pairs (Figure 1). In humans, there are 18 different α subunits and 8 different β subunits that together form 24 different, specific integrin heterodimers. Different integrin heterodimers bind different ligands, including RGD receptors, collagen receptors, laminin receptors, and leukocyte specific receptors (Hynes, 2002). Integrin expression is cell dependent and the different α/β subunit pairings formed dictate the types of external ligands that cells can bind, which has an influence on their differentiation and behavior.

Integrin structure and conformation are key to each part of its functionality, from adhesion to signaling. The α subunit has a head domain, upper and lower leg domains (Luo et al., 2007; Campbell and Humphries, 2011). Half of the α subunits have an I-domain inserted in the head domain, which allows them to coordinate divalent metal ions which can act as an activation switch (Luo et al., 2007; Campbell and Humphries, 2011). Much like the α subunit, the β subunit has upper and lower leg domains, and a head domain with a cation binding I-like domain (Luo et al., 2007; Campbell and Humphries, 2011). As a result of this structure, both α and β subunits are able to adopt conformations with bent and extended head groups (Luo et al., 2007; Campbell and Humphries, 2011). At inactive states, integrins are found in a bent conformation (Takagi et al., 2002; Luo et al., 2007; Ye et al., 2012).

The conformational change that allows the head group to swing out into an extended conformation correlates with activation (Takagi et al., 2002; Ye et al., 2012). Activation is a requirement for integrin binding to ligands and to mediate intracellular signaling. Upon activation, integrins have a higher affinity for ligand binding (Luo et al., 2007; Ye et al., 2012). Reflecting differences in ligand affinity for different conformations, integrins in the extended conformation are called active integrins, while integrins in the bent conformation are called inactive integrins. Though the extended head group is a hallmark of activated integrins, they can also adopt intermediate conformations such as an extended closed conformation where the headgroup is swung out but still maintains a low affinity for binding ligand (Ye et al., 2012; Su et al., 2016; Li and Springer, 2017).

### Sensing and Manipulating Integrin Activation Using Monoclonal Antibodies

Though changes in integrin activity are largely a result of ligand binding or recruitment of cytosolic scaffold proteins, activity state can also be manipulated or stabilized by specific monoclonal antibodies that are conformation sensitive and can be categorized by their functionality as either activating or blocking antibodies (Takada and Puzon, 1993; Byron et al., 2009; Campbell and Humphries, 2011; Su et al., 2016). Generally, activating antibodies promote ligand binding, while blocking antibodies prevent ligand binding (Byron et al., 2009). Though these two classes of antibodies have functional outcomes, the mechanism of action is different.

Blocking antibodies typically allosterically regulate the ligand binding site, stabilizing integrins in the inactive state and preventing ligand binding (Werb et al., 1989; Hall et al., 1990; Mould et al., 1996; Takagi and Springer, 2002; Byron et al., 2009; Su et al., 2016). The epitopes that many blocking antibodies bind are called Ligand Attenuated Binding Sites (LABS) and are often found very close to ligand binding sites (Humphries, 2004; Byron et al., 2009).

Activating antibodies are classified by the type of epitope they bind (Humphries, 2004; Byron et al., 2009). Ligand Induced Binding Site (LIBS) antibodies recognize epitopes that are only exposed when integrins are in the active conformation, though not necessarily only when ligand is bound, as the presence of cations (such as Mn^2+^) can induce this conformation and subsequent antibody binding (Bazzoni et al., 1995; Humphries, 2004; Byron et al., 2009). The binding of antibodies to the LIBS epitope stabilizes the integrin in the active state and increases the amount of ligand that can be bound by the integrins (Bazzoni et al., 1995; Luque et al., 1996; Byron et al., 2009; Su et al., 2016). By contrast, non-LIBS antibodies are activating antibodies that recognize epitopes that are exposed in a conformation independent fashion, and as a result can bind in the presence or absence of ligand (Tsuchida et al., 1997; Humphries, 2004; Byron et al., 2009). While LIBS antibodies stabilize the open conformation of integrins thus promoting ligand binding, the activation stimulated by non-LIBS antibodies likely primes the integrin for a conformation change to the active state in order to bind ligands (Humphries, 2004; Byron et al., 2009; Su et al., 2016). Interestingly, the non-LIBS activating antibody TS2/16 epitope partially overlaps with blocking antibodies AIIB2 and A1-A5, suggesting that functionally distinct epitopes are often in very close proximity (van de Wiel-van Kemenade et al., 1992). Because LIBS antibodies can only bind integrins that are already in the active conformation, they can both detect integrin activity state and promote a functional outcome, unlike non-LIBS or blocking antibodies that just regulate function.

### Roles for Divalent Ions and Disulfide Bonds in Integrin Conformation

Metal ion coordination in the I-domain of present in some α integrins plays a direct role regulating integrin activity by mediating changes in integrin conformation that increase binding affinity. On the other hand, metal ion coordination in the I-like domain of β integrins is less well defined and may be more important for the control of α/β heterodimers lacking an α I-domain, such as αvβ1 (Shimaoka et al., 2002; Takagi and Springer, 2002). As a result, changes in the extracellular concentration of divalent ions can promote the adoption of a specific conformation state. Excess Mg^2+^ and Mn^2+^ can displace Ca^2+^ within the I-domain and promote the adoption of open head group conformations and support ligand binding activity (Shimaoka et al., 2002; Takagi and Springer, 2002; Luo et al., 2007; Tiwari et al., 2011). Interestingly, when cells are treated with excess Mn^2+^ the affinity state achieved by compatible integrins is higher than that observed when integrins are activated by other means, providing further evidence for multiple different open integrin conformations (Luo et al., 2007; Ye et al., 2012). On the other hand, excess Ca^2+^ inhibits ligand binding, as it keeps integrins in a closed conformation (Takagi and Springer, 2002; Luo et al., 2007; Campbell and Humphries, 2011; Tiwari et al., 2011).

Integrin conformation and activity states can also be regulated by extracellular reducing agents acting on extracellular disulfide bonds of β subunits (Edwards et al., 1995; Yan and Smith, 2001; Chigaev et al., 2004; Ye et al., 2012). The addition of dithiothreitol (DTT) and to a lesser extent 2,3-dimercapto-1-propanesulfonic acid (DMPA) are able to stimulate integrin activity (Yan and Smith, 2001; Chigaev et al., 2004), in a manner independent from ion chelation (Edwards et al., 1995). DTT and DMPA appear to have an independent mechanism of activation from ion induced activation, as they can cause integrins to adopt multiple affinity states that occur at a much more gradual rate than the rapid activation of cation activation (Chigaev et al., 2004).

The importance of disulfide bonds also has been demonstrated when ligand binding was inhibited in response to treatment with N-ethylmaleimide, that blocks cysteines or the oxidizing agent phenylarsine oxide (Pujades et al., 1996; Edwards et al., 1998). N-ethylmaleimide treatment was noteworthy in that it prevented ligand binding to α4β7 (VLA-4) but still allowed cell-cell adhesion, meaning that these two integrin-mediated processes can be mediated by different integrin conformations (Pujades et al., 1996). Disulfide formation/reduction in α2β1 has also been linked to enhancing affinity to collagen (Lahav et al., 2003). Taken together these results provide supporting evidence for regulation of integrin function by redox circuits as a physiological control mechanism (Jones, 2008).

## Integrin Trafficking in Polarized Epithelial Cells

Most studies examining the trafficking of integrins have focused on endocytosis and assembly of focal adhesions as output variables, especially in migrating cells (Valdembri and Serini, 2012; Moreno-Layseca et al., 2019). Though it has been demonstrated that specific apical pools of integrins exist (Ojakian and Schwimmer, 1994; Garbi et al., 1996; McCormick et al., 1997), it is unclear whether there is an obligate intermixing of apical and basolateral integrins or whether they are independently regulated.

**FIGURE 2:**
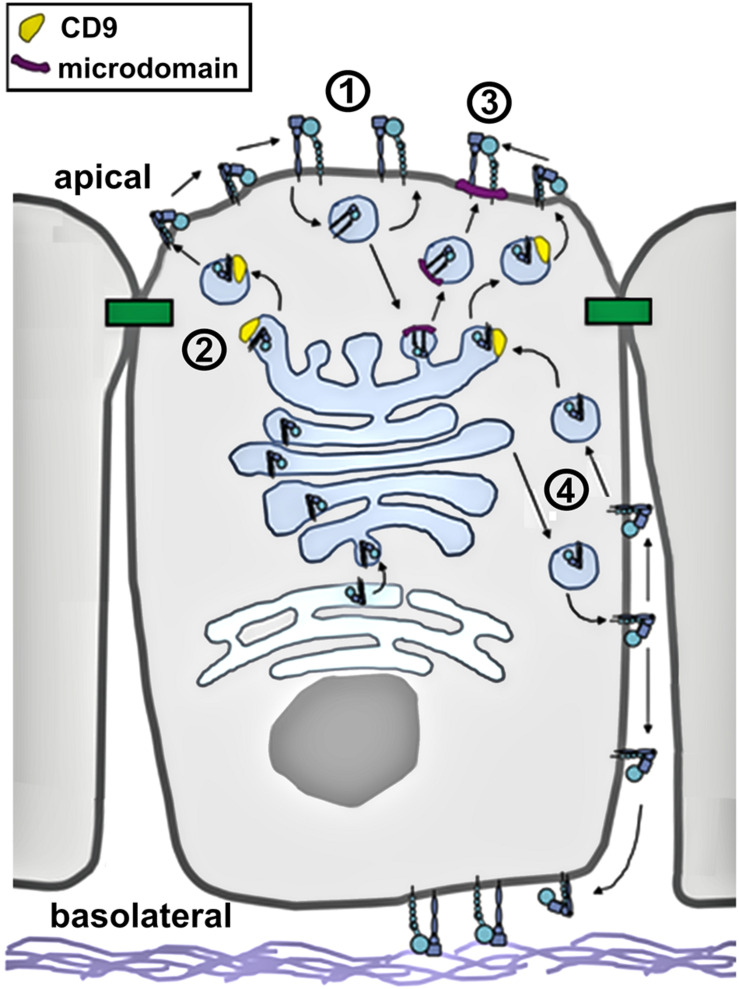
Integrin trafficking in polarized epithelia. In this diagram, putative trafficking pathways for active and inactive integrins are shown. These include (1) endocytosis followed by rapid recycling; (2) TM4SF/tetraspanin facilitated trafficking; (3) microdomain/raft mediated apical targeting, and (4) retrieval from the basolateral surface.

However, there are several clues from the current literature suggesting mechanisms that specifically localize them to the apical plasma membrane domain (Figure 2). The apical localization of integrins is mainly due to redistribution of the protein as opposed to resulting from increased expression of β integrin (Beaulieu, 1992; Hamzaoui et al., 2004).

Although the C terminal domains of alpha and beta integrins are short, they have a surprisingly diverse capacity to interact with different cytosolic regulatory proteins. Integrins have been found to directly bind to several clathrin adapter proteins, including GGA-2, GGA-3, and APPL1 (Ratcliffe et al., 2016; Diggins et al., 2018; Sahgal et al., 2019). The majority of endocytosed integrins are recycled through the *Trans* Golgi Network (TGN) (Moreno-Layseca et al., 2019) using a rab10/rab13 dependent pathway (Nokes et al., 2008), although direct recycling from sorting endosomes to the plasma membrane has also been demonstrated (Samarelli et al., 2020). In either case, recycling has the capacity to retain integrin localization to the apical domain. External stimuli such as galectin have the capacity to stimulate this integrin internalization and recycling pathway (Honig et al., 2018). Trafficking of integrins can also be state dependent. For instance, GGA-2 plays a role in regulating the internalization and recycling of active β1, but not inactive β1 (Sahgal et al., 2019). Cytosolic proteins, such as Vav3 have also been shown to regulate and stabilize apical localization of β1 integrin (Honig et al., 2018; Badaoui et al., 2020).

One clue to understanding the molecular regulation of integrin trafficking by epithelial cells comes from an examination of MDCK cells. MDCK cells deliver α5β1 to both the apical and basolateral domains, however, it is rapidly cleared from the apical surface, but stabilized on the basolateral surface (Gut et al., 1998). A key determinant for stabilization is the C terminal domain, which was found to be necessary and sufficient for accumulation of α5β1, based on integrin deletion mutants, which accumulated on the apical surface and Fc receptor chimeras containing integrin C-terminal domains, which were basolaterally retained (Gut et al., 1998).

The C terminus of both α5 and β1 contain NPxY motifs that are classically known as tyrosine-based internalization and sorting signals (Bonifacino and Traub, 2003). The role of NPxY motifs is more nuanced for integrin sorting in polarized cells, since they act as internalization signals for apically delivered integrins, but they are also critical to stabilize focal adhesion associated integrins by acting as binding sites for talin and kindlin (Reszka et al., 1992; Margadant et al., 2012; Elloumi-Hannachi et al., 2015).

Although integrins were shown to be delivered both apically and basolaterally in MDCK cells (Gut et al., 1998), this does not necessarily imply a lack of specific targeting. In fact, most proteins expressed by MDCK cells are specifically targeted either apically or basolaterally (Nelson and Yeaman, 2001), so the targeting of newly synthesized integrins to both the apical and basolateral surfaces is unusual. Relevant to this point, the tyrosine in the NPxY motif functions as a basolateral targeting signal for LDL receptors (Matter et al., 1994) and is thus likely to also target integrins to the basolateral plasma membrane.

Although the presence of a basolateral targeting motif could suggest that any apical delivery of integrins is non-specific, another possibility is that apical integrin delivery is mediated by a different pathway. A major pathway that targets the transport of newly synthesized protein and lipid to the apical surface is partitioning into cholesterol and sphingolipid enriched detergent resistant membrane microdomains (also referred to as lipid rafts) (Cao et al., 2012). There is considerable evidence that activated integrins partition into membrane microdomains (Lietha and Izard, 2020), suggesting a role for lipid rafts in sorting of active, open integrins to the apical plasma membrane. Consistent with a role for microdomains trafficking active integrins from the TGN to the apical surface, microdomains have also been shown to be involved in caveolar endocytosis of integrins (Cheng et al., 2006).

Apical targeting of newly synthesized, inactive, closed integrins may also involve membrane microdomains, but this is more likely to occur through an indirect pathway. It has been shown that inactive integrin β1 interacts with the tetraspanin proteins CD9, CD81 and CD151 and is transported to the apical aspect of intercellular junctions (Yanez-Mo et al., 2001). There is substantial broad literature demonstrating a role for tetraspanins in facilitating integrin trafficking in non-polarized cells (reviewed by Hemler, 2005). Since tetraspanins preferentially associate with gangliosides and cholesterol (Levy and Shoham, 2005), it is likely that trafficking of integrin/tetraspanin complexes would associate with the apical lipid raft targeting pathway, even though tetraspanins do not directly partition into classically defined lipid rafts (Hemler, 2005). In this model, the directional secretion of inactive integrins would be differentially targeted depending on whether they were associated with tetraspanins (apically directed) or not (basolaterally directed).

N-linked glycosylation also may play a role in targeting integrins to the apical surface. As one example, a significant portion of the apical pool of β1 was found to be enriched for N-linked glycans containing sialic acid which inhibits integrin β1 from binding fibronectin (Praetorius and Spring, 2002). Treatment with neuraminidase to remove sialic acid restored fibronectin binding. This suggests two possible modes of apical targeting, either directly through interaction with carbohydrate binding proteins (Fiedler et al., 1994; Chen et al., 2011) or by the retrieval of basloaterally delivered β1 that is inhibited from engaging the extracellular matrix.

## Function of Apical Integrins

**FIGURE 3:**
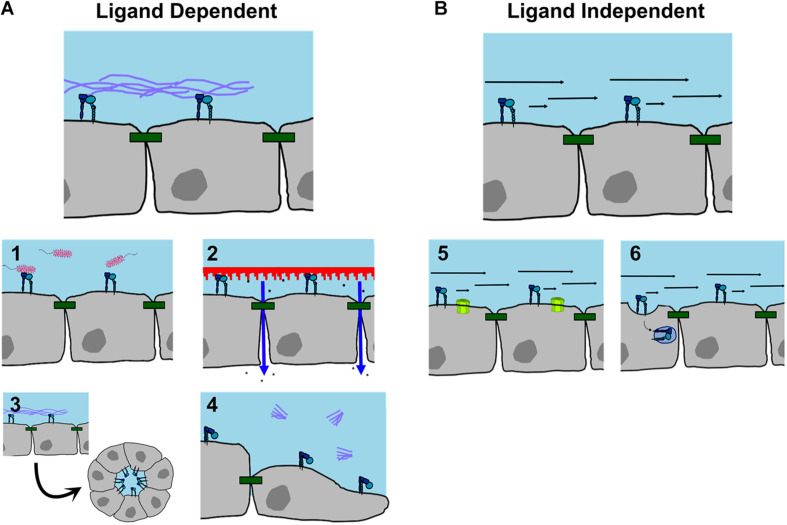
Functions of apically localized integrins. **(A)** Ligand dependent interactions. Apical integrins have been identified as (1) receptors for bacteria; (2) triggers increasing barrier permeability in response to substrate contact; (3) regulating apical/basolateral polarity and cyst formation; (4) promoting cell migration when blocked from ligand binding. **(B)** Ligand independent interactions. (5) Apical integrins can act as mechanosensors sensitive to fluid shear stress to open gap junction hemichannels; (6) or to induce caveolar endocytosis, which may regulate cell signaling. For references, see Table 1.

Despite the fact that most integrins localize to the basolateral or lateral surfaces of the cell, it is becoming well documented that integrins localize to the apical surface of the cell to serve functional roles (Table 1). Endogenous pools of apical integrins have been documented in many cell types and there are also examples of apical integrin localization induced by stimuli or cell phenotype. Many studies have identified β1 integrin as an apically oriented integrin, likely due to its prevalence and the reagents available for β1 labeling. Also, research frequently focuses specifically on β1 without considering specific alpha subunits associated with apical β1. Apically identified β1 containing heterodimers include α1β1, α2β1, α3β1, α5β1, α6β1 and αvβ1 (see Table 1 for references). Apical integrins are not restricted to β1, since αvβ5 (Mallavarapu and Finnemann, 2010; Uehara and Uehara, 2014), α6β4 (Frolikova et al., 2019), and β2 (Cinamon et al., 2004) have all been detected. Examples of stimuli associated with apical localization of integrins and their different functions are described below and summarized in Figure 3.

**TABLE 1:** Cells and Tissues with Apically Oriented Integrins.

**Cell/tissue**	**Integrins**	**Role**	**References**
**Kidney**			
MDCK cells	β1	Hypothesized role in wound healing	Praetorius and Spring, 2002
MDCK I cells	β1	Regulating tight junction associated actin, receptor for bacterial invasion	Tafazoli et al., 2000
MDCK II cells	α2β1, α3β1	Polarity, tubulocyst formation	Zuk and Matlin, 1996
	α5β1	Apical localization demonstrated	Gut et al., 1998
	β1	Apical localization demonstrated	Honig et al., 2018
K-ras transformed MDCK II cells	α2β1, α3β1	Cell migration/metastasis, Apical polarity defects	Schoenenberger et al., 1994
MDCK clone 8 cells	α2, α3, α6, β1	Polarity reversal	Ojakian and Schwimmer, 1994
Medullary collecting duct (rat)	β1	Hypothesized role in wound healing	Praetorius and Spring, 2002
**Intestine**			
Caco2 cells	β1	Receptor for bacterial invasion	Ragnarsson et al., 2008
	αvβ1	Increased transepithelial permeability	Walsh et al., 2015
	β1	Apical localization demonstrated, increased in hypoxia by HIF-1α	Zeitouni et al., 2016
	αvβ3	Targeted nanoparticles internalized, increased tight junction permeability	Xu et al., 2016
	α6, αv	Anionic nanoparticle binding increased tight junction permeability	Lamson et al., 2020
T84 cells	β1	Wound healing after neutrophil transmigration, increased tight junction leak	McCormick et al., 1997
M-like cells	α2β1	Apical localization demonstrated due to redistribution from basal surface	Hamzaoui et al., 2004
M cells (mouse)	β1	Hypothesized to be receptor for bacterial invasion	Clark et al., 1998
Ileum	β1	Receptor for bacterial invasion	Ragnarsson et al., 2008
	α2, α4	Hypothesized roles in cell/matrix and cell/cell interactions	Beaulieu, 1992
**Bone**			
Osteoblasts (rat)	β1	Bone growth/remodeling	Gohel et al., 1995
MLO-Y4 cells (mouse)	α5β1	Mechanosensing fluid flow shear stress opens Cx43 hemichannels	Batra et al., 2012
**Lung**			
Bronchial airway (mouse), primary airway epithelial cells, Calu-3 cells	β1	CFTR knockdown and Fdel508/Fdel508 genotype increases apical β1 expression, associated with increased ceramide/sphingosine ratio, impaired bacterial clearance	Grassme et al., 2017; Badaoui et al., 2020
Lung tissue (rat)	αvβ3	Apical localization demonstrated in lung endothelium	Singh et al., 2000
**Eye**			
Lens epithelial cells (mouse)	α6β1	Mechanosensing fluid flow shear stress opens Cx50 hemichannels	Liu et al., 2020
Lens epithelium (chicken)	α3β1	Epithelial to mesenchymal transition	Zuk and Hay, 1994
Retinal pigment epithelial cells	αvβ5	Hypothesized roles in ligand stabilization and photoreceptor outer segment fragment endocytosis	Mallavarapu and Finnemann, 2010
	α5β1	Hypothesized roles in attachment to neuroretina, photoreceptor outer segment fragment endocytosis, cell migration	Li et al., 2009
**Endothelium**			
Aortic Endothelial Cells (bovine)	β1	Mechanosensing fluid flow shear stress, may activate TRPV4 channels	Yang and Rizzo, 2013
HUVECs	α2β1	Hypothesized role in angiogenesis	Turner et al., 2020
	β2	High levels of apical integrin allow neutrophil migration to take place independent of shear signals. Activation state of integrin matters	Cinamon et al., 2004
Splenic sinus endothelial cells (rat)	αvβ5	Co-localized near vesicles hypothesize they play a role in endocytosis	Uehara and Uehara, 2014
**Other**			
FRT thyroid cells (rat)	α1β1	Collagen binding causes polarity reversal, increases tight junction leak	Garbi et al., 1996
Mammary tissue (mouse)	α2β1	Tissue development, growth, proliferation	Keely et al., 1995
F98 glioma cells	β1	Regulation of cell migration/metastasis	Mang et al., 2020
NCI-N87 gastric carcinoma cells	α5β1	Apical localization demonstrated	Feige et al., 2018

### Reproduction and Control of Cell Phenotype

There is abundant evidence that apical pools of integrins, particularly β1, play important roles in reproduction, particularly in implantation (reviewed in Sueoka et al., 1997; Wang and Armant, 2002; Kaneko et al., 2011). Integrins α3β1, α6β1, and α6β4 have been identified on the apical surface at the head of sperm where they play roles during acrosomal exocytosis and help mediate initial interactions with the egg surface (Frolikova et al., 2019). In blastocysts and endometrial cells, the apical localization of integrin β3 has been determined to be crucial for embryo implantation (Kaneko et al., 2011), and apoptosis of follicular cells in the ovary (Meehan et al., 2015).

As one example of differential integrin localization associated with tissue phenotype, expression levels of α2β1 were found to change during mammary tissue differentiation, as does apical integrin localization (Keely et al., 1995). There is apical localization of α2 integrin during maturation and puberty, but it decreases during pregnancy and lactation, suggesting a role for apical integrins in mammary tissue growth and proliferation associated with the ability to produce milk (Keely et al., 1995).

Application of a type I collagen hydrogel to the apical surface of human umbilical vein endothelial cells (HUVECs) induces the reorganization of α2β1 integrins to the apical surface of the cells (Turner et al., 2020). It was further demonstrated that the activation of α2β1 integrins was an important step in rapid tube formation and angiogenesis (Turner et al., 2020).

Schoenenberger, et al. demonstrated that MDCK II cells that have been transformed by K-*ras* expression and exhibit apical polarity defects also have apical localization of α2β1 and α3β1 integrin (Schoenenberger et al., 1994). MDCK cells were also used to identify that apical integrins are a definitive feature for the formation of tubulocysts as blocking collagen type I, type IV, and laminin interactions with integrin β1 by using AIIB2 blocking antibodies prevents the formation of tubulocysts all together (Ojakian and Schwimmer, 1994; Zuk and Matlin, 1996), as does the AJ2 blocking antibody (Ojakian and Schwimmer, 1994). Interestingly the study by Zuk and Matlin shows that the addition of collagen overlay on the apical surface of the cell does not have an effect on the relative size of apical pools of β1 integrin (Zuk and Matlin, 1996). This suggests that the presence of apical integrins themselves does not have an effect on polarity, but rather interactions between apical integrins and external ECM components promote a reorientation of the apical/basolateral polarity axis (Ojakian and Schwimmer, 1994; Zuk and Matlin, 1996). This was evidenced by the loss of gp135 at the membrane (Ojakian and Schwimmer, 1994; Zuk and Matlin, 1996), the random redistribution of p58 at the membrane (Zuk and Matlin, 1996), and the loss of microvilli (Ojakian and Schwimmer, 1994) after exposure to apical collagen overlays.

In rat osteoblasts, β1 integrins are localized equally to apical and basolateral surfaces, but treatment with IGF-I, which stimulates linear bone growth, causes a significant increase of the β1 integrin localized to the apical surface (Gohel et al., 1995). Likewise, when osteoblasts are treated with corticosterone, which is known to inhibit bone growth in part by blocking IGF-I production, there is a decrease of integrin subunits on both the apical and basolateral surfaces, suggesting that the apical localization of integrins may play a role in regulating bone growth (Gohel et al., 1995).

### Apical Integrins as Bacterial Receptors

Several bacteria have been shown to invade cells by binding to integrins as opportunistic receptors, either directly or indirectly through an interaction with ECM proteins (Scibelli et al., 2007; Hoffmann et al., 2011). Among the best characterized of these pathogens is the interaction between *Yersinia* and apically localized integrin β1.

The infection of epithelial cells by *Yersinia* is due to adhesion mediated by bacterial protein invasin binding to integrin β1 (McCormick et al., 1997; Clark et al., 1998; Tafazoli et al., 2000). The highest risk for infection is thought to be after neutrophil migration to the monolayer where access to integrin β1 at the basolateral surface is highest (McCormick et al., 1997). However, high rates of neutrophil transmigration in columnar intestinal epithelial cells have been demonstrated to allow for apical localization of integrin β1, likely due to disruption of the apical junctional complex. Interestingly, pools of apical integrins remain even after the epithelial barrier is restored (McCormick et al., 1997).

In T84 intestinal cells, apical integrins alone do not increase susceptibility to bacterial infection (McCormick et al., 1997), however, apical integrins appear to be sufficient for increased infection in studies using MDCK and Caco-2 cells (Tafazoli et al., 2000). These studies showed bacterial adhesion to apically localized β1 integrin subunits was crucial in disrupting barrier function, assembly of TJ proteins, and opening the barrier to make the basolateral integrins more accessible to the bacteria (Tafazoli et al., 2000). The proximity of apical β1 integrin to cell junctions seemingly played an important role in bacterial infection as they provided a spot for bacterial adhesion adjacent to the junctional proteins that are targeted by bacterial cytotoxins (Tafazoli et al., 2000). Interestingly, there is evidence that *Yersinia* infection in both Caco-2 cells and human ileal tissue promotes an increase in apical localization of integrin β1 (Ragnarsson et al., 2008).

As a potential way to target integrin mediated infection, the apical localization of β1 integrin in intestinal epithelial cells has been shown to be regulated by oxygen tension, since hypoxia decreases apically localized β1 leading to decreased internalization of *Yersinia* (Zeitouni et al., 2016). The decrease in apical β1 integrin was associated with an increase in HIF-1α. Cells that were treated with dimethyloxalylglycine, a HIF-1α stabilizing agent, also had reduced apical localization of β1 integrins, suggesting that HIF-1α might be a regulator of integrin expression and localization at either a transcriptional or post-translational level (Zeitouni et al., 2016).

### Regulation of Epithelial Barrier Function

The current standard of care for transdermal delivery of large molecular weight biologics (such as etanercept) is bolus injection. An improved delivery device was recently developed consisting of a microneedle array drug delivery device, where the microneedles are coated with a nanostructure imprinted polyether ethyl ketone (PEEK) film that is critical to enhance transepithelial drug delivery (Walsh et al., 2015; Aldrich et al., 2017; Kwon et al., 2019). It was demonstrated *in vitro* that contact between nanostructured PEEK and αvβ1 integrins at the apical surface of Caco-2 cell monolayers increases epithelial permeability as measured by both decreased TER and increased paracellular flux of macromolecules (Kam et al., 2013; Walsh et al., 2015; Stewart et al., 2017; Huang et al., 2020). Paracellular leak induced by nanostructured films was accompanied by a hallmark change in tight junction morphology from a linear to ruffled appearance (Lynn et al., 2020), as evidenced by ZO-1 immunofluorescence. Nanostructure stimulation was MLCK-dependent, associated with changes in organization of the actin cytoskeleton, and also induced changes in the integrin associated protein talin (Walsh et al., 2015; Huang et al., 2020). Apically added RGD peptides further mimicked the effects of nanostructures on epithelial barrier function, which supports a role for integrins in regulating tight junctions (Walsh et al., 2015).

Garbi et al. demonstrated that application of collagen overlays to rat thyroid monolayers resulted in a decrease in TER, loss of E-cadherin basal polarity, and the formation of a cystic structure where the cells reoriented to form a lumen (Garbi et al., 1996). This is attributed to apical pools of α1β1 integrins, since blocking anti-β1 integrin with antibodies prolonged the time it took to see a decrease in TER (Garbi et al., 1996).

Studies in Caco-2 cells have demonstrated that chitosan coated nanoparticles bind to apically localized αvβ3 integrin, which leads to an opening of tight junctions and loss of junction associated ZO-1 and claudin-4 (Xu et al., 2016). Anionic silica nanoparticles have also been shown to increase paracellular leak to macromolecules and decrease TER by interacting with α6 or αv integrin to stimulate an MLCK-dependent permeability pathway (Lamson et al., 2020). Whether integrin-stimulated reorganization of tight junctions by particles is strictly due to apical integrins specifically regulating the cytoskeleton or whether it also requires nanoparticle internalization remains an open question.

### Regulation of Cell Migration

It is well established that integrins in contact with the ECM can directly regulate cell migration, but there are several examples where integrins on the upper cell surface indirectly regulate cell migration.

Retinal pigment epithelial (RPE) cells are enriched with both αvβ5 and α5β1 integrins that are localized to the apical surface of the cell (Li et al., 2009; Mallavarapu and Finnemann, 2010). While there is evidence that these integrins play roles attaching RPE cells to the neuroretina and during photoreceptor outer segment fragment endocytosis, they are also involved in RPE migration after wound healing (Li et al., 2009). Antagonizing α5β1 in RPE cells prevented cell migration and proliferation, possibly as a result of disrupting F-actin and ZO-1 organization (Li et al., 2009).

K-*ras* transformation of MDCK II cells caused the apical localization of pools of α2β1 and α3β1 integrin (Schoenenberger et al., 1994). K-*ras* activation has been shown to regulate the overall both integrin expression levels and play a role in cell invasion (Schramm et al., 2000; Hirata et al., 2018). While there is no direct evidence that K-*ras* transformation driven apical localization of integrins is involved in metastasis, it is an interesting consideration. Non-transformed MDCK cells also share some similar characteristics with developing and mechanically damaged kidney epithelium, so the fact that apical β1 integrins have been identified in these cell monolayers and can bind fibronectin suggests that apical localization of integrins plays a role in development and/or wound healing (Praetorius and Spring, 2002). On the other hand, enhanced apical localization of collagen binding integrins, such as α3β1, have been associated with epithelial to mesenchyme transition (Zuk and Hay, 1994) and might act as a switch associated with a cancer phenotype as opposed to productive wound healing.

Integrins are highly dynamic and traffic from the bottom surface to the top in rapidly migrating cells through a pathway where they are internalized and recycled to the bottom of the cell at the leading edge (Gu et al., 2011). This pool of integrins is also likely to have a signaling function, since integrins on the top surface of human lung and skin fibroblasts has been shown to bind fibronectin (Akiyama et al., 1989). Disruption of integrin ligand binding with blocking antibodies to either α5 or β1 decreases cell adhesion and promotes cell migration (Akiyama et al., 1989). Consistent with this model, integrins on the upper surface of migrating skin fibroblasts are highly mobile, as measured by FRAP, whereas they are clustered into structures referred to as fibrillar streaks in stationary cells (Duband et al., 1988).

Consistent with this observation, when apical pools of β1 integrins on F98 cells are in the closed conformation, the cells are highly migratory (Mang et al., 2020). However, when cells are treated with ligand that both clusters and activates the apical β1 subunits there is a reduction of focal adhesions and cell elongation that in turn inhibits migration (Mang et al., 2020). Taken together, these studies suggest that engagement between apical integrins and specific ligands provides a molecular switch that regulates cell motility.

### Mechanosensing

In lens epithelial tissue, studies have shown α6β1 integrin localized to the apical surface (Liu et al., 2020). When the α6β1 heterodimer in lens epithelial tissue is activated by fluid flow shear stress, it causes the opening of Cx50 hemichannels as a pathway to enable metabolite permeation into this avascular tissue (Liu et al., 2020). The activation of β1 integrin by activating antibody TS2/16 causes a similar opening of Cx50 hemichannels, but this activation appears to be entirely dependent on α6 participation, as blocking α6 prevents opening of Cx50 by either activating β1 antibody or fluid flow shear stress. This suggests that there are mechanosensing roles for apical integrins that are ligand independent. Moreover, Cx50 and α6 co-immunoprecipitated, indicating they were part of a multimeric complex that required motifs present in the C terminus of Cx50 (Liu et al., 2020).

A similar pathway has been demonstrated in osteocytes, where the α5β1 heterodimer opens Cx43 hemichannels under conditions of mechanical force that cause flow of interstitial fluid (Batra et al., 2012). As was the case in lens cells, treatment of osteocytes with the activating antibody TS2/16 caused a force independent opening of Cx43 and α5 formed a precipitable complex with Cx43 (Batra et al., 2012).

The vasculature is also sensitive to mechanical stimulation due to blood flow. Consistent with a role for integrins in sensing flow, bovine aortic endothelial cells (BAECs) have apically localized β1 integrins that respond to shear stimulation (Yang and Rizzo, 2013). Interestingly, the apical β1 integrin response to shear stress in BAEC cells was actin-independent and was instead associated with caveolae and eNOS stimulation, which distinguishes it from integrins mediating cell adhesion to the ECM (Yang and Rizzo, 2013).

β2 integrin at the apical surface of HUVECs also regulates leukocyte translocation in a tunable manner. At basal levels of expression, β2 integrin mediates leukocyte adhesion, however, transmigration requires the added mechanical stimulus of flow or chemokine activation (Cinamon et al., 2004). However, high levels of activated β2 integrin expressed at the apical surface of HUVECs enables neutrophil transmigration in the absence of flow, which might contribute to excessive inflammation (Cinamon et al., 2004).

## Signal Transduction by Apical Integrins

To date, it seems most likely that molecular complexes used to induce cell signaling by apical integrins are equivalent those used by integrins in general. However, apically localized integrins differ from ECM localized integrins in ways that can influence their physiologic function. Apical integrins are not often assembled into immobile focal adhesions and thus may be more rapidly reconfigured in response to different stimuli than ECM engaged integrins. In addition, the function of apical integrins is almost certainly influenced by the local microenvironment of the apical plasma membrane, which differs from the basolateral plasma membrane. They also have access to apical ligands, including ECM components that are apically secreted, most notably fibronectin.

### Complex Formation

Clustering of apical integrins, particularly activated integrins, appears to be a key component in inducing cell responses ranging from cell migration to increases in permeability (Ojakian and Schwimmer, 1994; Garbi et al., 1996; Zuk and Matlin, 1996; Kam et al., 2013; Walsh et al., 2015; Grassme et al., 2017; Stewart et al., 2017; Badaoui et al., 2020; Mang et al., 2020; Turner et al., 2020). Apical integrin clustering can also drive endocytosis which can be physiological, in the case of integrin turnover during cell migration, but also has the pathological consequences of facilitating infection by bacteria using apical integrins as receptors.

How clustering of apical integrins is linked to integrin conformation and activation is not well defined. Several studies have demonstrated that integrin activation, usually through ligand binding (Ojakian and Schwimmer, 1994; Garbi et al., 1996; Zuk and Matlin, 1996; Grassme et al., 2017; Badaoui et al., 2020; Mang et al., 2020; Turner et al., 2020), but not necessarily clustering (Xu et al., 2016) were necessary to cause cells to respond. Recent evidence demonstrates that β1 integrins associated with focal adhesions contain nanoclusters with distinct populations of both active and inactive integrins suggesting two independent pools depending on activation state (Spiess et al., 2018). Given the transition from freely mobile integrins to clustered, immobile integrins that is associated with cells becoming stationary (Duband et al., 1988), it is likely that clustering and integrin activation state are interconnected at the apical surface as well. Taking advantage of tools such activation state sensitive antibodies, novel approaches to specifically promote integrin clustering and super-resolution imaging techniques will help define mechanisms by which apical integrins influence cell function.

### Regulation of Actin

Much like their basolaterally localized counterparts, apically localized integrins interact with the actin cytoskeleton (Tafazoli et al., 2000; Li et al., 2009; Yang and Rizzo, 2013; Uehara and Uehara, 2014; Walsh et al., 2015; Badaoui et al., 2020; Mang et al., 2020). One possible explanation for the differential roles for apical and basal integrins is that the apical integrins access unique pools of actin that are distinct from actin interacting with integrins associated with focal adhesions. For instance, Bisaria et al. (2020) identified a membrane proximal pool of F-actin, and if apical integrins mainly access this pool of actin this could explain why apical integrins are involved with processes such as apical signal transduction, mechanosensing, and regulation of barrier function. Identifying specific pools of actin that are preferentially regulated by apical integrins will help refine hypotheses linking them to the regulation of other actin-binding proteins, such as tight junction associated ZO-1 (Belardi et al., 2020).

Critical to understanding how apical integrins interact with the actin cytoskeleton is to determine how scaffold proteins may be recruited by stimulation of apical integrins. Currently, there is a paucity of information on how stimulating apical integrins affect interactions with scaffold proteins like kindlin, vinculin, or focal adhesion kinase, although there is indirect evidence showing a reorganization of talin in response to stimulation with apically applied nanostructured surfaces (Huang et al., 2020).

In addition to the actin cytoskeleton, apical integrins have also been linked to changes in plasma membrane lipid composition, enriching ceremide and diminishing sphingosine (Grassme et al., 2017). Likewise, data linking caveolin, eNOS and connexin hemichannels to apical integrin mediated mechanosensing (Batra et al., 2012; Yang and Rizzo, 2013; Liu et al., 2020) expands the scope of integrin-interacting proteins beyond actin and classical integrin scaffold proteins.

### Apical Integrin Ligand Engagement

Basolateral integrins are classically linked to establishment of the apical/basolateral polarity axis by a signaling cascade involving Rac1, basolateral laminin secretion and integrin-linked kinase signaling (Lee and Streuli, 2014; Matlin et al., 2017). However, apical integrins can also serve as cues to influence epithelial cell polarity. Many studies defining roles for apical integrins in driving reversal of apical/basolateral polarity relied on substrate overlay techniques to stimulate apical integrins (Ojakian and Schwimmer, 1994; Garbi et al., 1996; Zuk and Matlin, 1996; Walsh et al., 2015; Turner et al., 2020). While these techniques demonstrate that clustering of apical integrins has a functional outcome, they are difficult to interpret as integrin-specific because the substrate has contact with the entire apical surface of the cells likely leading to stimulation of other receptors. Increased permeability and tight junction reorganization seen in cells treated apically with nanostructured surfaces are subject to a similar complication (Kam et al., 2013; Walsh et al., 2015; Stewart et al., 2017). Based on inhibitor experiments and expression profiling, the effects of apical substrate overlay on epithelial cells were found to require myosin light chain kinase and focal adhesion kinase (Walsh et al., 2015), however, Rac1 expression decreased (Kam et al., 2013), suggesting that signaling induced by stimulation of apical integrins partially differs from the pathways used by basolateral integrins engaged to an established ECM. Whether the key basolateral factor integrin-linked kinase plays a role in apical integrin signaling is an open question.

A more specific approach to stimulate apical integrins was used by Turner, et al., who showed that targeting apical integrins with antibody coated polystyrene beads replicates tube formation in a way comparable to collagen hydrogel exposure (Turner et al., 2020). This suggests that at least in some situations, apical integrin stimulation alone is sufficient to induce the same results seen with a substrate overlay technique.

The studies described above used experimentally placed ligands to stimulate apical integrin signaling. There are few reports examining apical secretion of ECM proteins, however, there is evidence that the endogenous β1 ligand fibronectin is apically secreted. Constitutive apical fibronectin secretion is measurable, but lower than basolateral secretion and varies with cell type (Low et al., 1994). This contrasts with laminin and collagen that are almost exclusively basolaterally secreted, although there is one report that injured kidney epithelial cells show increased apical collagen secretion (Sauvant et al., 2005).

Several stimuli that regulate fibronectin secretion include TGF-β1 (Wang et al., 1991) and adenosine (Walia et al., 2004) which increase apical secretion and progesterone which decreases apical secretion (Mularoni et al., 1992). Proteolytic cleavage of the ECM is another means by which the apical pool of fibronectin has been shown to be increased (Badaoui et al., 2020). Stimulation of apical fibronectin secretion by TGF-β1 is accompanied by a change in mRNA splicing leading to a change in fibronectin isoform expression (Wang et al., 1991). Whether different fibronectin isoforms are apically targeted or whether splicing and targeting are independently regulated has not been determined. Nonetheless, an increase in apical secretion of fibronectin in response to TGF-β1 stimulation is likely to have as yet undetermined roles for embryonic development and wound repair, particularly as part of a polarity reversal pathway that can induce epithelial to mesenchymal transition (Gonzalez and Medici, 2014).

## Physiologic Consequences of Apical Integrin Signaling

Many physiological roles for pathways mediated by apical integrins are relatively straightforward, such as regulation of cell migration as a response to wound repair and formation of epithelial cysts and tubes as a mechanism of development. A link between integrin mechanosensing and connexin hemichannel opening is important for providing nutrients and removing waste products from avascular tissues (bone, lens) (Batra et al., 2012; Liu et al., 2020). The ability to increase epithelial barrier permeability by stimulating apical integrins has been well established, and has utility as a means to promote drug delivery (Walsh et al., 2015; Stewart et al., 2017; Lamson et al., 2020), however, the physiologic context and mechanisms of action for this property of apical integrin signaling is still under investigation.

One of the most studied pathologic consequences of apical integrin expression is in the disease cystic fibrosis (CF). Studies in transgenic mouse models expressing mutant cystic fibrosis transmembrane regulator (CFTR) have shown that CF bronchial epithelial cells are enriched with β1 integrin at the luminal surface, a characteristic absent in bronchial epithelial cells from wild type mice (Grassme et al., 2017). An examination of primary human airway epithelial cells has also confirmed the enrichment of apical β1 integrin in cells from CF patients (Badaoui et al., 2020). Apical localization of β1 integrin in CF has been linked to increased Vav3 expression which stabilizes integrin localization at the plasma membrane. Increases in apically localized β1 integrin in CF were associated with increased bacterial infection through multiple pathways, including disruption of sphingolipid metabolism leading to a decrease in sphingosine-mediated bacterial killing (Grassme et al., 2017). Moreover, increased apical β1 enhances airway cell adhesion of bacteria such as *Staphylococcus* (Hoffmann et al., 2010) and *Pseudomonas* (Gagniere and Di Martino, 2004; Badaoui et al., 2020; Thuenauer et al., 2020), which has the potential to facilitate biofilm formation and bacterial endocytosis, worsening patient morbidity. These observations provide several potential therapeutic targets with the capacity to improve CF patient condition by enhancing the ability to combat bacterial infections. It is also likely that changes in apical integrin expression may play roles in the pathological consequences of other diseases beyond CF, which will likely be revealed by future research.

## Future Directions

In considering the vast integrin literature, there has been a consistent undercurrent of evidence supporting the apical localization and function of integrins that complements their more traditional roles as ECM receptors and in mediating cell-cell interactions. However, there are many open questions that need to be considered. For instance, there have been a handful of *in vivo* studies focusing on apical integrin function (Walsh et al., 2015; Grassme et al., 2017; Wu et al., 2018; Liu et al., 2020) as compared with the majority of the literature which uses cultured cell models.

Whether some functions of apical integrins, such as mechanosensing, are truly ligand independent remains an open question, and could be resolved by identifying novel ligands. Also, many studies have identified involvement of apical integrins in different cell processes and that they are related to regulation of the cytoskeleton. But there is often a gap in understanding how apical integrin-dependent regulation of actin is linked to other downstream targets.

The signal transduction pathways stimulated by apical integrins have not been fully determined and it is likely that there will be significant differences between apical and basolateral integrin signaling. In addition, integrins classically thought of as being in an inactivated conformation in the context of ECM engagement may prove active when present on the apical plasma membrane surface and in the right context, such as an appropriately clustered state. The ability to analyze the fine structure of apical integrin complexes in relation to other cell components in native settings using super-resolution microscopy is one potentially fruitful approach to identifying novel non-classical roles for integrins in regulating cell function.

## Author Contributions

Both authors conceived, wrote and edited the manuscript, contributed to the article, and approved the submitted version.

## Conflict of Interest

The authors declare that the research was conducted in the absence of any commercial or financial relationships that could be construed as a potential conflict of interest.

## Publisher’s Note

All claims expressed in this article are solely those of the authors and do not necessarily represent those of their affiliated organizations, or those of the publisher, the editors and the reviewers. Any product that may be evaluated in this article, or claim that may be made by its manufacturer, is not guaranteed or endorsed by the publisher.
